# Transforming single-band static FSS to dual-band dynamic FSS using origami

**DOI:** 10.1038/s41598-020-70434-y

**Published:** 2020-08-17

**Authors:** Akash Biswas, Constantinos L. Zekios, Stavros V. Georgakopoulos

**Affiliations:** grid.65456.34 0000 0001 2110 1845 ECE, Florida International University, Miami, 33199 USA

**Keywords:** Electrical and electronic engineering, Electronic and spintronic devices

## Abstract

Frequency selective surfaces (FSSs) have been used to control and shape electromagnetic waves. Previous design approaches use complex geometries that are challenging to implement. With the purpose to transform electromagnetic waves, we morph the shapes of FSS designs based on origami patterns to attain new degrees of freedom and achieve enhanced electromagnetic performance. Specifically, using origami patterns with strongly coupled electromagnetic resonators, we transform a single-band FSS to a dual-band FSS. We explain this transformation by showing that both symmetric and anti-symmetric modes are excited due to the strong coupling and suitable orientation of the elements. Also, our origami FSS can fold/unfold thereby tuning (i.e., reconfiguring) its dual-band performance. Therefore, the proposed FSS is a dynamic reconfigurable electromagnetic structure whereas traditional FSSs are static and cannot change their performance.

## Introduction

Origami is the art of paper folding that can transform planar sheets to 3D geometries, and it has recently inspired scientists and engineers in different disciplines. Using its geometrical and structural properties, innovative designs of mechanical metamaterials have been introduced throughout the last 10 years^1–20^. For instance, Eidini et al.^1^ studied the mechanical properties and origami folding techniques of a specific pattern (i.e., Miura-Ori), showing its applicability to a wide range of applications from mechanical metamaterials to deployable structures. Hongbin et al.^7^ showed that by using the self-locking and reconfiguration mechanisms of origami patterns, mechanical metamaterials with programmable properties can be developed. Boatti et al.^8^ obtained a wide range of thermal expansion coefficients by using origami metamaterials. By using origami topologies, Fang et al.^10^ successfully constructed various types of two and three dimensional Bravais lattices that can undergo through diffusion-less phase transformations when they are rigidly folded. Treml et al.^11^ used an origami waterbomb as an experimental platform to demonstrate a 1-bit mechanical storage device that writes, erases and rewrites itself in response to a time-varying environmental signal. Miura^21^ introduced the concept of large membranes in space (deployable solar panel), thereby proving the applicability of origami patterns and providing new mechanical performance. These previous works have clearly demonstrated that origami patterns can alter the performance of structures and provide new mechanical performance.

Similarly, reconfigurable electromagnetic structures based on origami geometries have also been introduced and extensively explored^22–26^. In electromagnetic structures, the phenomenon of reconfiguration is well known from antennas to filters. By changing an antenna’s length, shape or spacing of its radiating elements, we can alter its resonant frequency, radiation pattern, and other EM characteristics^27,28^. Le et al.^18^ investigated a programmable metamaterial based on ternary foldable origami in the gigahertz regime, providing four transformable modes corresponding to four different functions of electromagnetic reflector and frequency-selective absorbers. Liu et al.^22,26^ investigated origami bifilar and multi-radii monofilar helical antennas that can operate in different frequency bands. Yao et al.^24^ designed a two-arm Nojima origami conical spiral antenna that can morph from a planar dipole to a conical spiral. Nauroze et al.^23^ designed an origami structure loaded with electrical components that can be reconfigured over continuous-state ranges from folded to unfolded configurations. Fuchi et al.^25^ studied the transmission characteristics of a folded surface loaded with a periodic arrangement of split ring resonators.

Frequency selective surfaces (FSSs) are spatial filters comprised of periodically organized electromagnetic elements that are strongly coupled. The type of element used in a FSS design defines its performance^27^. Many sophisticated techniques have been used to appropriately guide and control acoustic waves^29^, and electromagnetic waves in both the microwave^30–39^ and the photonic^40,41^ regimes. Zhou et al.^29^ presented a multi-band double negative acoustic metamaterial based on coupled Helmholtz resonators that exhibits multiple alternatively double-positive and double-negative passbands. Ferreira et al.^30^ achieved a dual-band frequency selectivity, in which the upper resonance frequency can be tuned independently of the first one. Phon et al.^31^ presented a multifunctional active frequency selective surface based on the switching responses of active components. Kern et al.^33^ introduced a multiband artificial magnetic conductor design using FSS screen that have fractal or nearly fractal unit cell geometries. Ranjbar et al.^34^ achieved a wide range of polarization transformations over broad bandwidths, as well as multiple bands by cascading subwavelength dielectric gratings. Cheng et al.^35^ proposed a near-perfect dual-band circular polarizer based on bi-layer twisted, split-ring resonators. Xu et al.^36^proposed a two-layer chiral metamaterial inspired from fractals, which forms chirality over triple bands. Lin et al.^37^ proposed a dual-band and high efficiency reflective cross-polarization converter based on an anisotropic metasurface for linearly polarized electromagnetic waves. Kim et al.^38^ proposed a new family of multilayer impedance-matched chiral metasurfaces that offer arbitrary polarization control at two different frequencies. Londoño et al.^39^ proposed a special class of Huygen’s surfaces that is able to manipulate transmitted wave fronts, while exhibiting high transparency over a broad range of frequencies. As it can be observed from the aforementioned works, it is highly important to develop FSS designs that operate in multiple frequency bands. Even though all these works^30–39^ have presented novel FSSs, their designs exhibit high complexity. It is indeed very challenging to design FSSs of low complexity that operate in multiple frequency bands and exhibit low fabrication costs. To address this challenge, a novel FSS is introduced here by combining origami mathematics with electromagnetics.

Origami FSSs have been already introduced^23,42–46^. Fuchi et al.^42,43^ introduced foldable frequency selective surfaces that can be tuned by changing their folding states. Specifically, in^42^ dipoles were printed on Miura-Ori tessellations showing a resonance shift up to $10\%$ when their inter-element distances were changing by folding. Also, in^43^ a similar study was performed using Jerusalem cross resonators that showed a resonance shift up to $19\%$ of the resonant frequency. Sessions et al.^44^ also examined origami inspired FSSs focusing on the dependence of the operational frequency and polarization on folds. Recently, in^45^ the authors designed an origami FSS to maintain its frequency response across multiple fold angles. Finally, Nauroze et al. used additive manufacturing techniques to design^23,46^ multilayer FSSs on Miura-Ori patterns that exhibited increased bandwidth compared to single-layer FSSs. These published works on origami FSSs, have primarily studied only the effects of folding on their performance. Here we reveal and explain an electromagnetic phenomenon that occurs in our proposed origami FSS. Our analysis shows that origami transforms a single-band static FSS to a dynamic dual-band FSS by conforming periodically spaced electromagnetic resonators to a Miura-Ori folding pattern, thereby transforming a traditional single-band FSS to a dual-band one. This is an important finding as it proves that by conforming the shape of electromagnetically coupled elements to origami patterns, we can create new transformations of EM waves and develop new FSS designs with enhanced electromagnetic performance. This phenomenon is independent of the frequency of operation and can be observed in microwaves, photonics and optics; therefore, it can be used in various applications across the available electromagnetic spectrum. To demonstrate our findings, a loop resonator is used in the origami FSS examined here. This FSS is simulated, fabricated and experimentally tested. Finally, the proposed origami FSS, as it is expected, is a dynamic reconfigurable structure since it can fold/unfold thereby tuning its dual-band performance depending on its folding state.

## Phenomenon explanation

**Figure 1:**
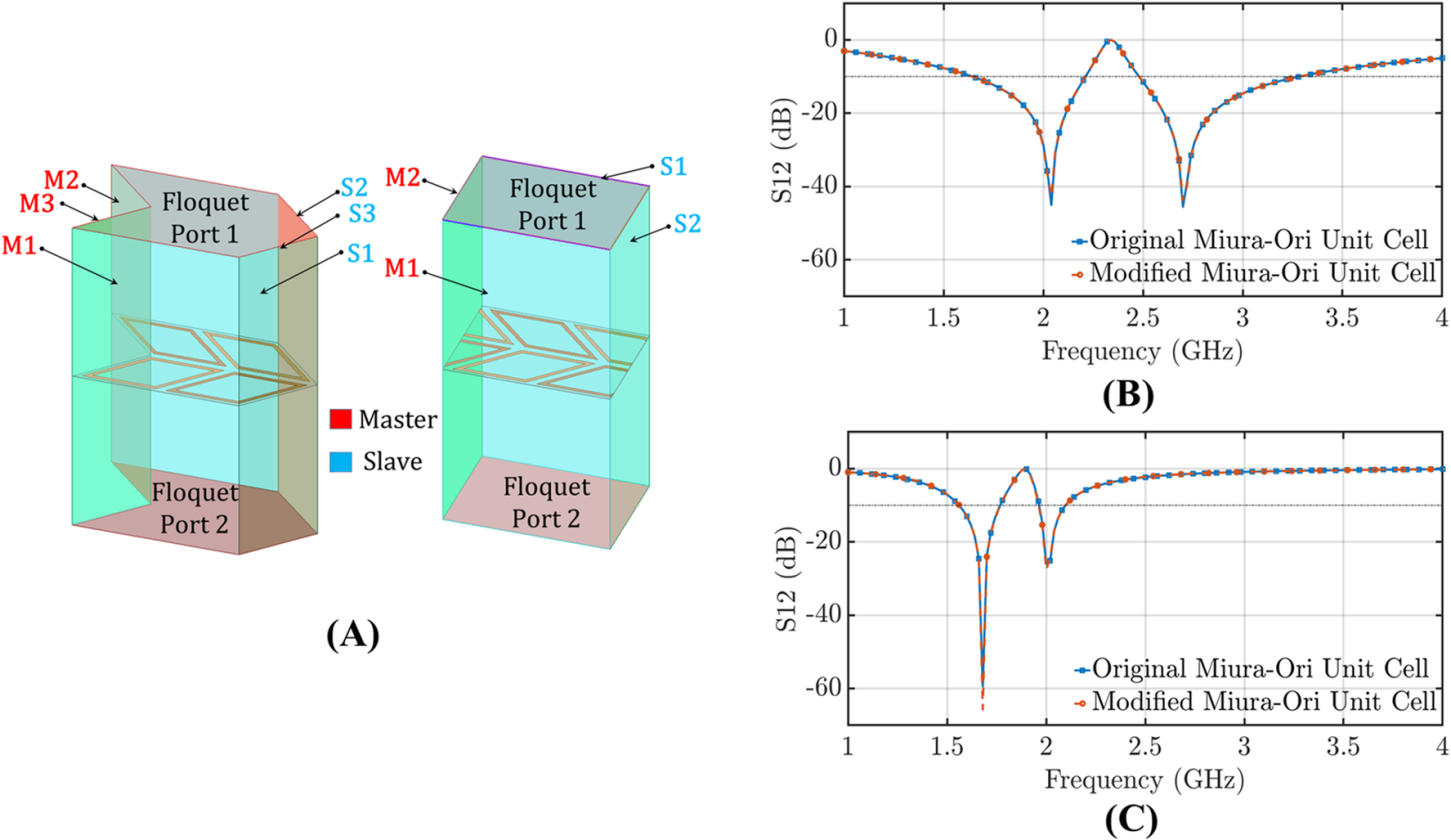
(**A**) Numerical models with Floquet ports; On the left, three Master/Slave boundary conditions (original Miura-Ori unit cell). On the right two Master/Slave boundary conditions (modified Miura-Ori unit cell). Transmission coefficient for the two different configurations of our unit cell for: (**B**) TE polarization. (**C**) TM polarization.

Full-wave EM analysis is performed using ANSYS HFSS to compare the responses of the standard FSS and the proposed origami FSS. To save computational time and resources, since the geometry under study is a periodic structure only the unit cell is simulated. Unlike previous analyses^23^, the unit cell is appropriately defined so that we minimize the computational cost of the simulation. Instead of defining a unit cell of 3 periodic boundary surfaces (Fig. 1A left), we define a modified unit cell (Fig. 1A right) with only two periodic surfaces emulating the 2*D* periodicity of our design.

**Figure 2:**
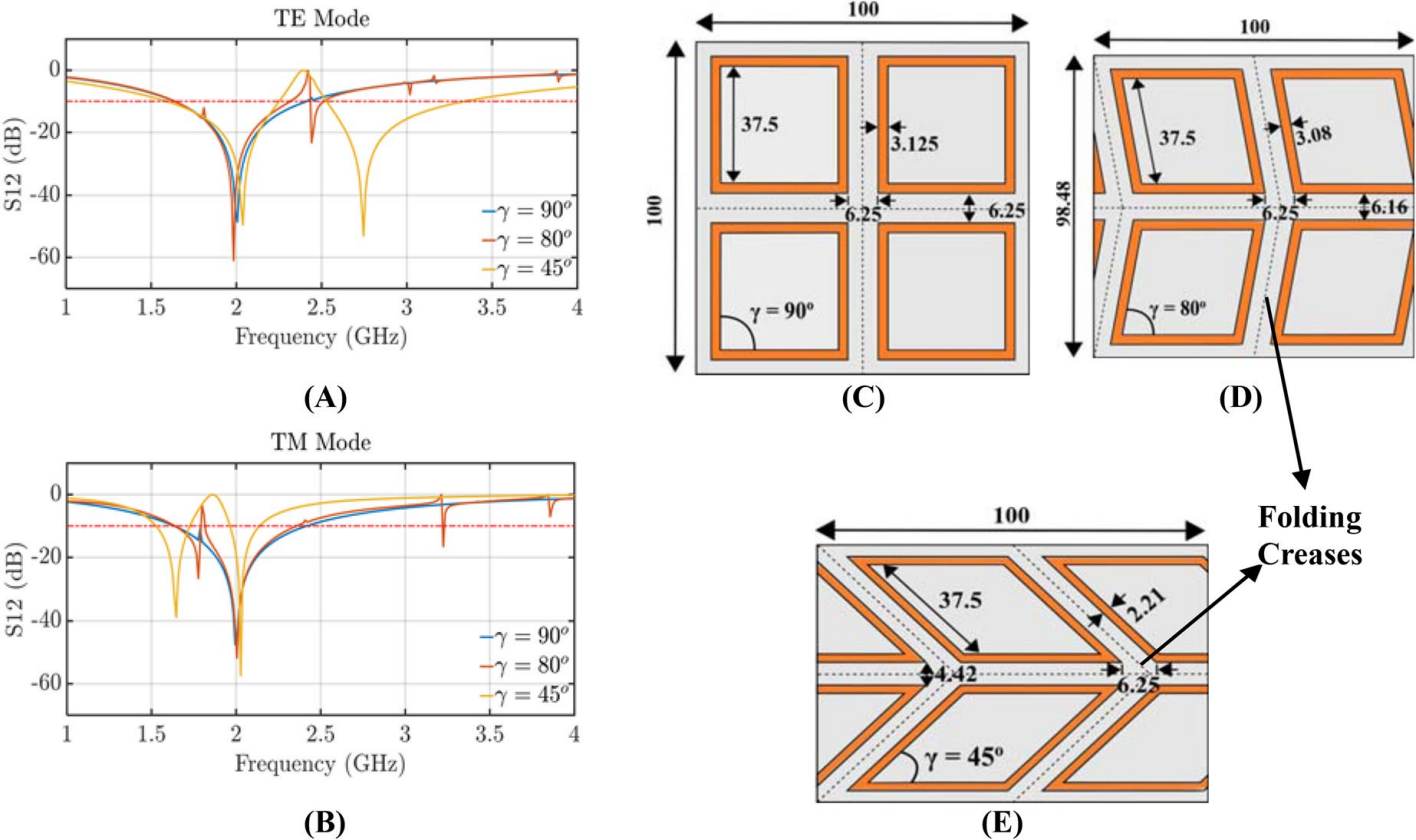
(**A**) Transmission coefficient for TE polarized incident waves; as the relative angle between the elements changes a second resonance appears. (**B**) Transmission coefficient for TM polarized incident waves; as the relative angle between the elements changes a second resonance appears. (**C**) Unit cell of standard square loop FSS. (**D**) Proposed origami FSS unit cell with distorted square loops that conform to the Miura-Ori design $\gamma = 80^{\circ }$. (**E)** Proposed origami FSS unit cell with distorted square loops that conform to the Miura-Ori design $\gamma = 45^{\circ }$.

This modification of the studied unit cell accelerates the simulation process 3 times (Table 1), in the case where a workstation of $2\times$Intel Xeon silver 4114 Processors (10 cores, 13.74 MB Cache, 2.20 GHz) and 256 GB (DDR4, 2,666 MHz ECC RDIMM) is used. Figure 1B,C show that the transmission coefficient is identical for the two different configurations of our unit cell. To demonstrate the effect of the origami pattern on the periodic arrangement of the resonators, different Miura-Ori topologies loaded with ring resonators are modeled as shown in Fig. 2.

**Table 1:** Computational demands for the simulation of the original and modified Miura-Ori unit cell, respectively.

Simulation setup	Computational time (h)	Memory demand (GB)
6 sided rhombic-loop FSS unit cell (original Miura-Ori unit cell)	3	6.23
4 sided rhombic-loop FSS unit cell (modified Miura-Ori unit cell)	1	5.6

**Figure 3:**
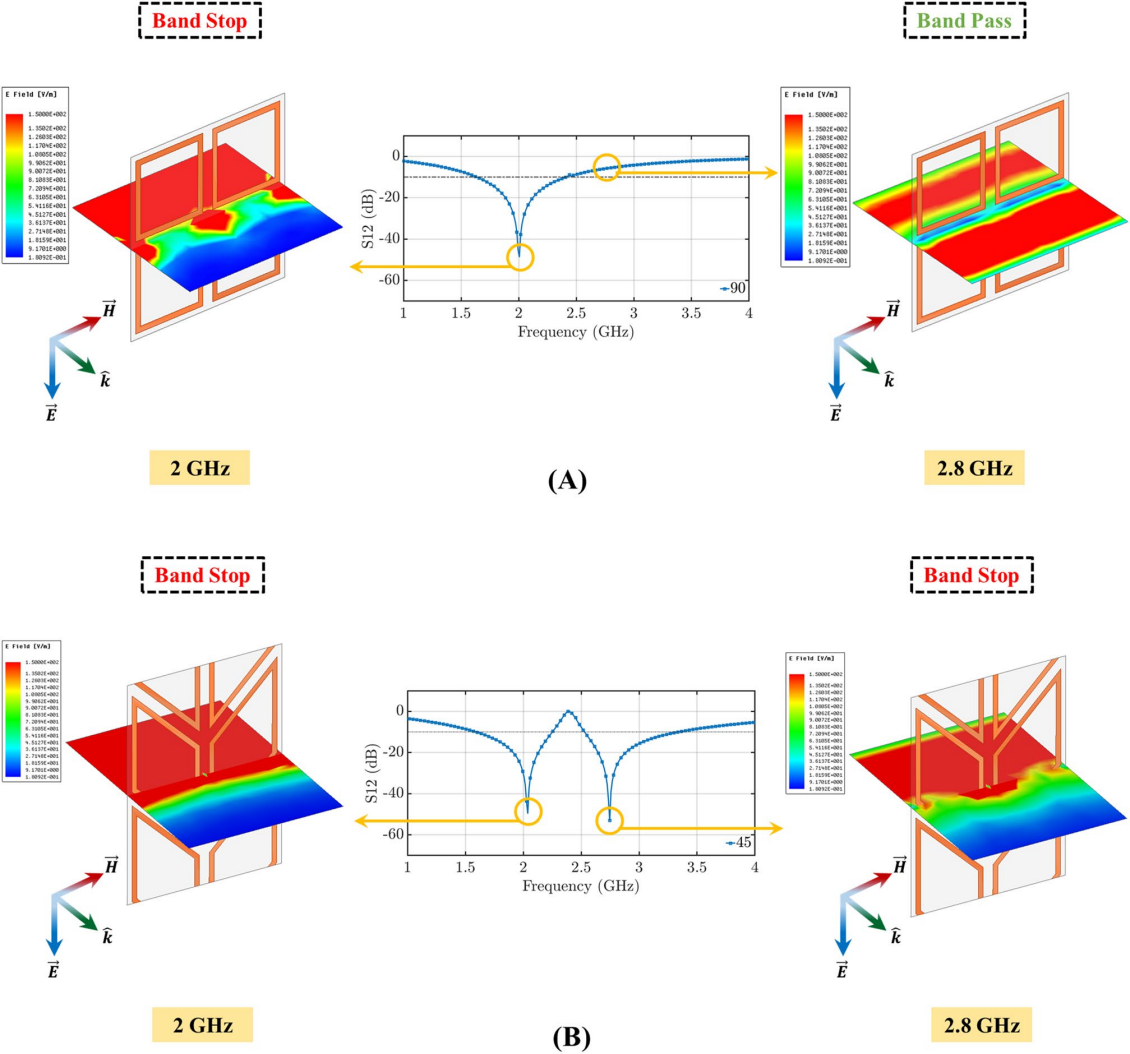
Electric field distribution for TE mode excitation for: (**A**) $\gamma = 0^{\circ }$ corresponding to standard square loop FSS, which exhibits single-band filtering performance and (**B**) $\gamma = 30^{\circ }$ corresponding to the proposed origami FSS, which exhibits dual-band filtering performance.

**Figure 4:**
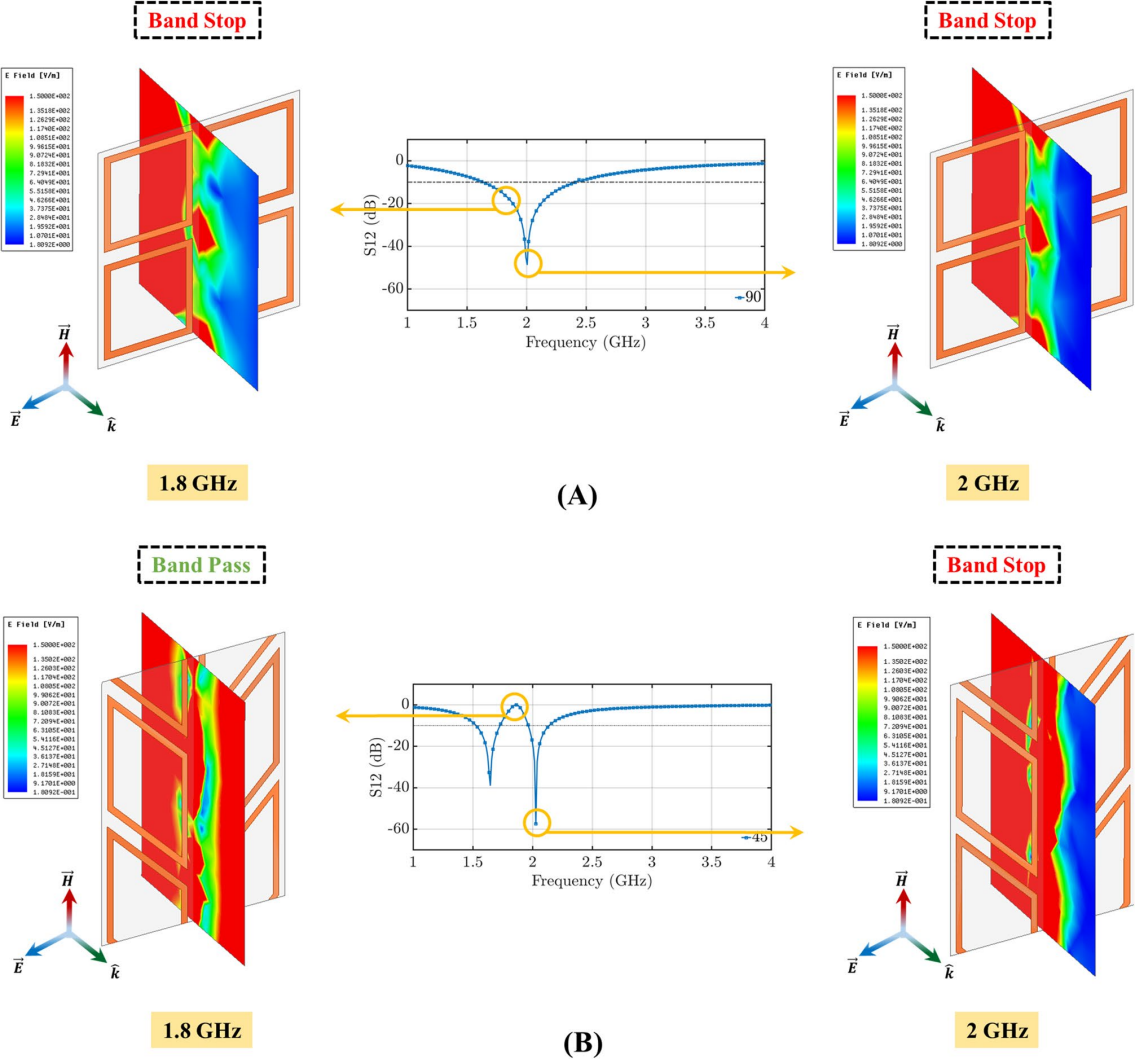
Electric field distribution for TM mode excitation for: (**A**) $\gamma = 0^{\circ }$ corresponding to standard square loop FSS, which exhibits single-band filtering performance and (**B**) $\gamma = 30^{\circ }$ corresponding to the proposed origami FSS, which exhibits dual-band filtering performance.

Figure 2A,B illustrate the simulated transmission coefficients of the standard FSS (the unit cell of its geometry is shown in Fig. 2C), and two example designs of the proposed origami FSS (the unit cells of their geometries are shown in Fig. 2D,E. The responses in Fig. 2A,B show that for both TE and TM polarizations a single-band FSS is transformed into a dual-band one by conforming the standard square-loop resonator (corresponding to the case with $\gamma =90^{\circ }$) to the Miura-Ori pattern (corresponding to the cases with $\gamma \ne 90^{\circ }$; specifically, the two example cases of $\gamma = 80^{\circ }$ and $\gamma = 45^{\circ }$ are shown in Fig. 2). Figures 3 and 4 confirm this by comparing the electric field distributions of the origami FSS with distorted loops ($\gamma =45^{\circ }$) and the standard FSS with square loops ($\gamma =90^{\circ }$) . Specifically, Fig. 3A shows that the standard FSS with square loop filters the incident field at only 2 GHz, whereas the origami FSS filters the incident field at both 2 GHz and 2.8 GHz, for a TE incident wave. Similarly, Fig. 4A shows that the standard FSS with square loop has only one stop band and filters the incident field at both 1.8 GHz and 2 GHz, whereas the origami FSS produces two distinct stop band frequency ranges by filtering the incident field at 2 GHz and 1.6 GHz and one band pass frequency range around 1.8 GHz for TM incident wave.

In general, it is well known that when the square loop resonator, or any rhombus, is placed in a periodic FSS configuration, where all the elements and their sides are parallel to each other, a single resonant behavior is achieved^27^. This resonance is independent of the shape of the resonator and is identical with the resonance of the square loop (Fig. 2C), which corresponds to the blue curves in Fig. 2A,B, when $\gamma =90^{\circ }$. Also, the shape of the loop only affects the polarization response of the impinging wave for different angles of incidence. However, our origami FSS design (see two example cases for $\gamma = 80^{\circ }$ and $\gamma = 45^{\circ }$ in Fig. 2D,E and their corresponding transmission coefficients in Fig. 2A,B) achieves for the first time a dual-band performance by orienting the resonators so that they conform to the Miura-Ori pattern (see Supplementary Movie 1 Online). Figure 2D,E show that for these two cases the sides of the loops are parallel to the folding lines of the Miura-Ori pattern.

**Figure 5:**
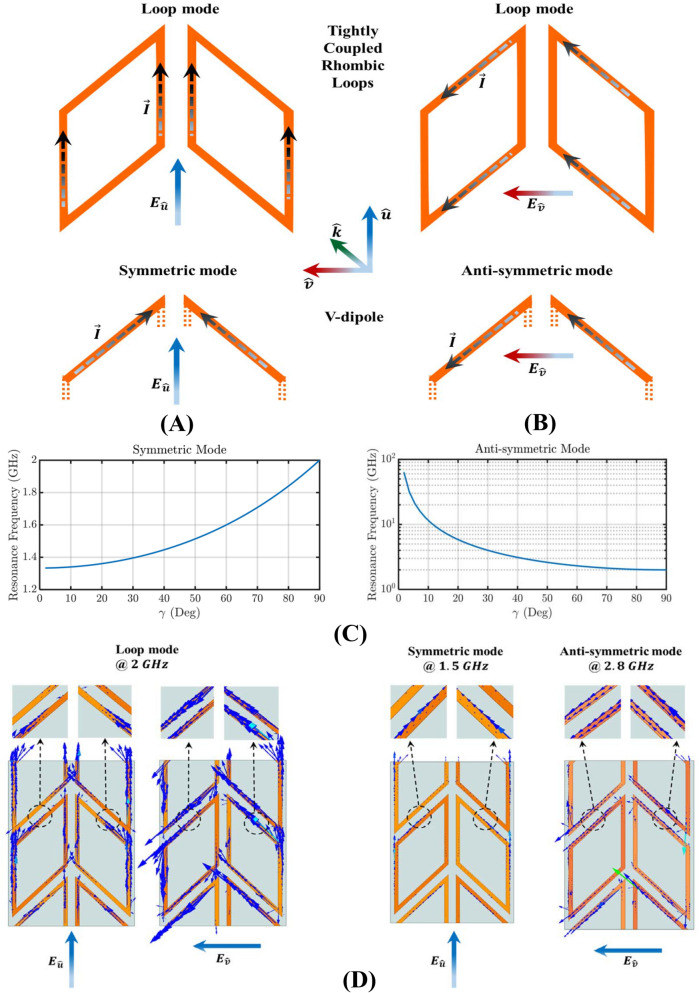
(**A**) Current distribution for an $\vec {E_u}$ impinging wave that excites both the first-order loop mode and the symmetric V-dipole mode of the two tightly-coupled rhombic loops. The small distance between the rhombic loops allows their slanted sides to strongly couple and create a V-dipole configuration. (**B**) Current distribution for an impinging wave that excites both the first-order loop mode and the anti-symmetric V-dipole mode of the two tightly-coupled rhombic loops. The small distance between the rhombic loops allows their slanted sides to strongly couple and create a V-dipole configuration. (**C**) Resonant frequency dependence of the V-dipole in terms of the angle $\gamma$ for an $\vec {E_u}$ impinging wave (left) and a $\vec {E_v}$ impinging wave (right), respectively. (**D**) Current distributions of the first-order loop modes and symmetric and anti-symmetric V-dipole modes based on eigenanalysis at the corresponding frequencies. The current intensities of the dominant loop modes are as expected larger than the current intensities of the V-dipole modes.

To understand why this happens, we isolate two tightly coupled loops and we define two unit-vectors to describe their orientation: $\vec {u}$ along the symmetry axis of the two shapes and $\vec {v}$ perpendicular to $\vec {u}$ , as shown in Fig. 5A,B. In this configuration, these two elements behave as coupled loops and as coupled V-antennas^47,48^. Depending on the polarization of the electromagnetic field, symmetric and anti-symmetric modes are excited.

To analyze the EM behavior of our FSS, eigenvalue analysis is performed to provide physical insight^49^. The tightly-coupled rhombic loops support two eigenmodes of opposite symmetry, which are excited simultaneously with the fundamental eigenmodes of the typical uncoupled loop. Thus, an incident electric field, which is parallel to the axis of symmetry of the coupled loops (i.e., $\vec {E_u}$), excites both the V-dipole symmetric mode and the loop mode, as shown in Fig. 5A. Also, an incident field, which is perpendicular to the axis of symmetry (i.e., $\vec {E_v}$), excites both the V-dipole anti-symmetric mode and the loop mode, as shown in Fig. 5B. In summary, depending on the field orientation, the symmetric/antisymmetric and loop modes are simultaneously excited. For a loop with side length, *h*, (e.g., $h=37.5\,{\hbox {mm}}$ in Fig. 2), a loop resonance is observed when the circumference is equal to one wavelength, i.e., $\lambda _{loop}= 4h$, where $\lambda _{loop}$ is the effective wavelength^28^. In addition, depending on the orientation of the field, the symmetric and anti-symmetric modes are observed. In the anti-symmetric mode, the current distribution at each arm approximates that of an individual antenna of length $\lambda _{anti{\text {-}}sym}=4sin(\gamma )\cdot {h}$ for the case of an $\vec {E_v}$ impinging wave (see Fig. 5C right). In the symmetric mode, the current distribution at each arm approximates that of an individual antenna of length $\lambda _{sym}=4(cos(\gamma )/2+1)h$ for the case of an $\vec {E_u}$ impinging wave (see Fig. 5C left). Figure 5C shows the frequency dependence of the V-dipole in terms of the angle $\gamma$ for the TE and TM polarizations. For example according to the equations mentioned, for the case of $\gamma =45^{\circ }$, the loop mode resonates at $f_{loop}\simeq 2$ GHz, the symmetric mode at $f_{sym}\simeq 1.5$ GHz and the anti-symmetric mode at $f_{anti-sym}\simeq 2.8$ GHz, respectively, which coincide with the results of the full-wave simulation as shown in Fig. 2A,B. Figure 5D also shows the current distributions of the tightly coupled loops of Fig. 2E based on our eigenanalysis, which prove the existence of symmetric, anti-symmetric, and loop modes at the expected frequencies.

**Figure 6:**
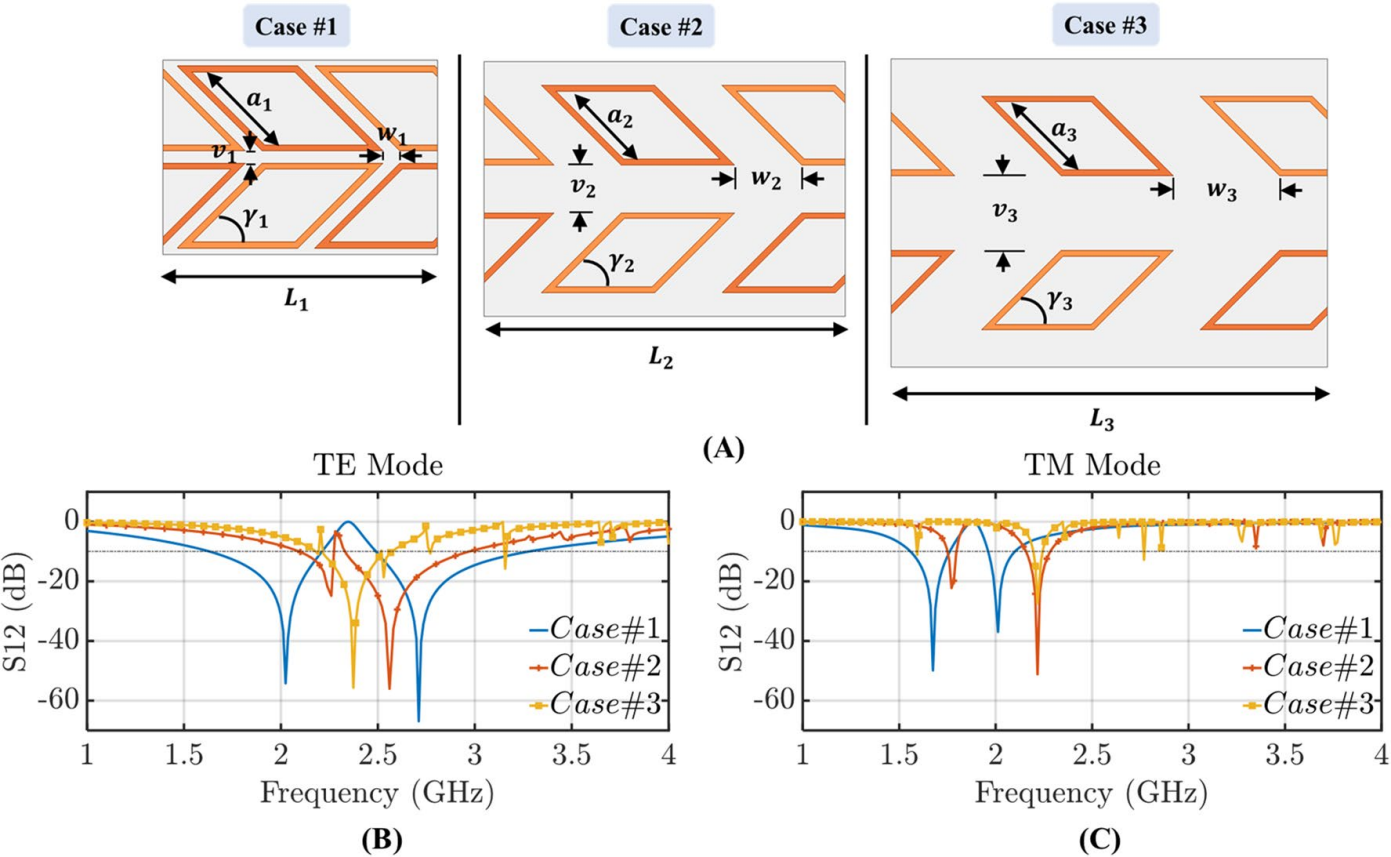
(**A**) Proposed origami FSS unit cell with distorted square loops ($a_1=a_2=a_3=37.5\,{\hbox {mm}}$ and $\gamma _1$ = $\gamma _2$ = $\gamma _3 = 45^{\circ }$) that conform to the Miura-Ori for different inter-element spacing. (**B**) Transmission coefficient for TE polarized incident waves for cases #1, #2, and #3 with $w_1=0.04\lambda$, $w_2=0.14\lambda$ and $w_3=0.27\lambda$, respectively. (**C**) Transmission coefficient for TM polarized incident waves for cases #1, #2, and #3 with $v_1=0.04\lambda$, $v_2=0.17\lambda$ and $v_3=0.34\lambda$, respectively.

**Figure 7:**
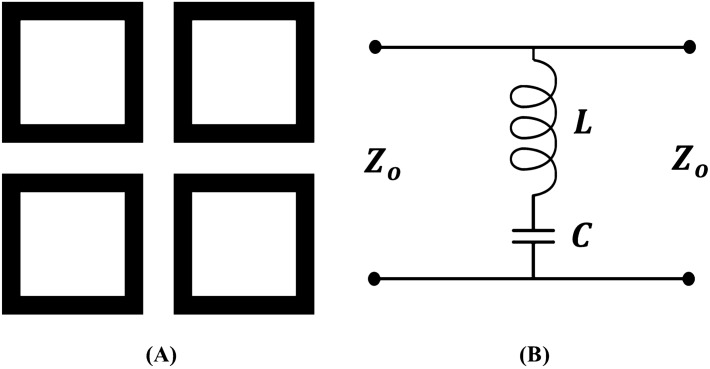
(**A**) Typical square loop resonator. (**B**) Equivalent circuit model of square loop resonator.

As shown above by using the origami pattern and utilizing the strong coupling of the rhombic resonators we transform a single band FSS to a dual-band FSS. A simulation study is performed to examine the effects of the coupling between the resonators to the dual-band response. In this study, the size of the resonators is kept constant (aiming to achieve the same frequency band of operation) and the gap between them is varied, as shown in Fig. 6. Essentially, as the gaps between the resonators become wider, the resonators become smaller compared to the facets. As it is shown in Fig. 6A as the resonators move further apart from case #1 towards case #3 the FSS forms a single stopband for both its TE (Fig. 6B) and TM (Fig. 6C) polarizations. Specifically, this transition from dual-band to single-band performance occurs when the distance between the resonators is $\sim \lambda /3$, where $\lambda$ is equal to the circumference of one loop and as a wavelength corresponds to a frequency of 2 GHz. Also, along with the dual- to single-band transformation a frequency shift is observed for our band of operation. This frequency shift is expected since as the gap between the resonators widens, their mutual capacitance decreases. This can be easily understood using the equivalent circuit model of a typical square loop resonator that has been extensively studied in^27,50,51^ and is shown in Fig. 7 for completeness.

## Experiments of a Miura-Ori FSS

**Figure 8:**
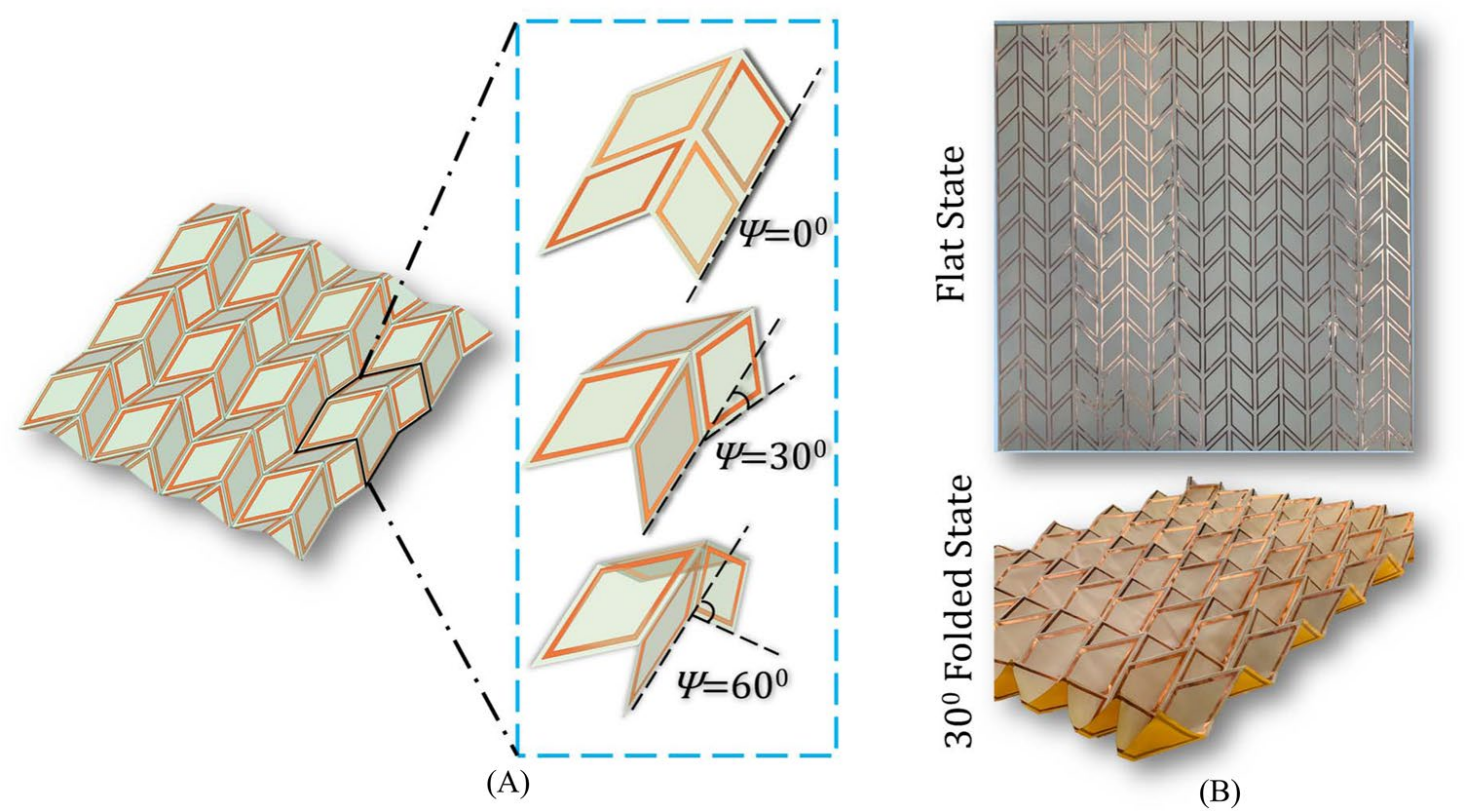
Rhombic loop FSS on a Miura-Ori geometry. (**A**) $4\times 3$ FSS film in an arbitrary folded angle showing the unit cell at different folding states. From top to bottom: flat unit cell at folded angle $\psi =0^{\circ }$, folded unit cell at folded angle $\psi =30^{\circ }$. and folded unit cell at folded angle $\psi =60^{\circ }$. (**B**) Fabricated FSS on polyimide substrate in its flat state (top) and folding state for $\psi =30^{\circ }$ (bottom).

For an experimental demonstration, we fabricated an FSS with conductive rhombic loops that conform to a Miura-Ori design of $45^{\circ }$ unit-cell pattern (Fig. 8). The prototype was constructed using traditional printed circuit board (PCB) fabrication on a $60\times 56.6\,{\hbox {cm}}^2$ polyimide substrate of relative permittivity $\epsilon _r = 3.4$, relative permeability $\mu _r= 1$ and thickness of 50 µm (Fig. 8B).

**Figure 9:**
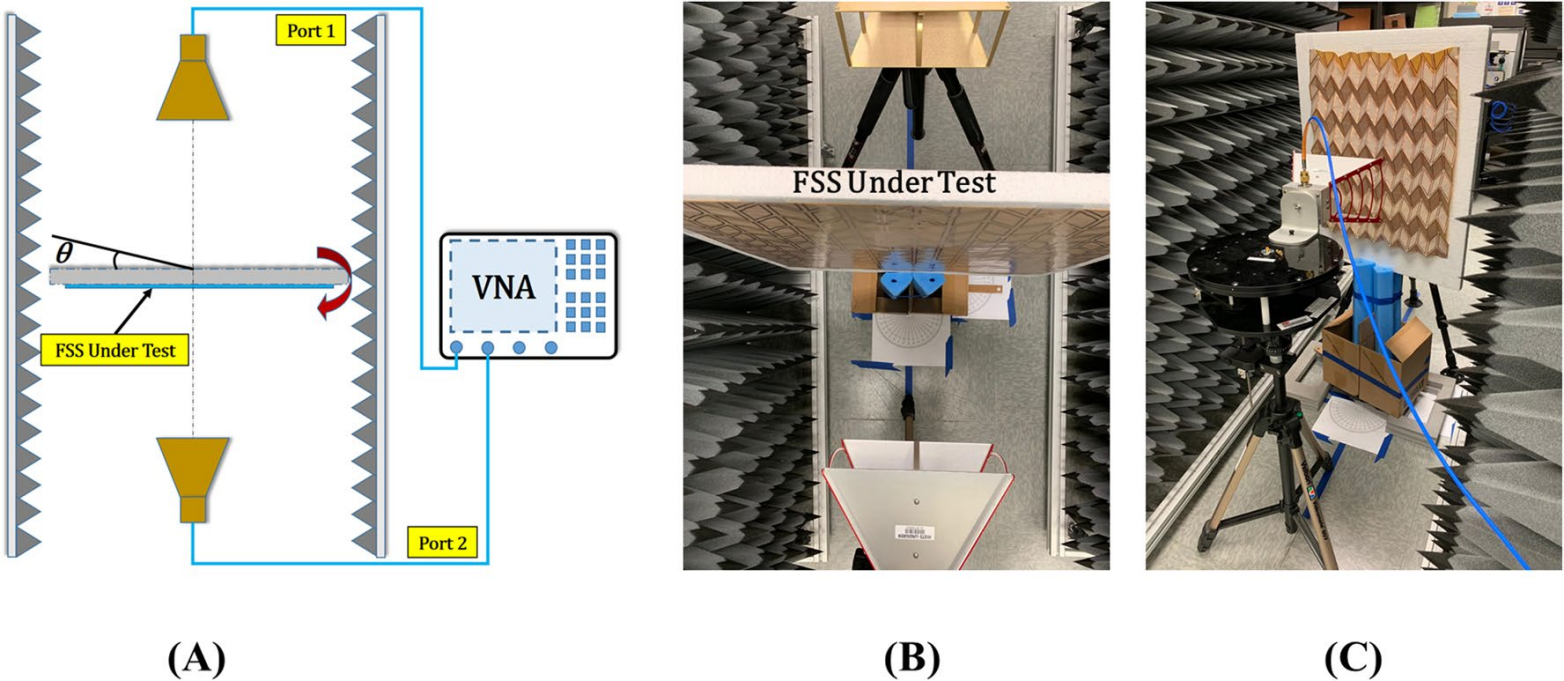
Measurement setup of the proposed origami FSS. (**A**) Schematic diagram of the setup with two absorbing walls left and right, and two wideband horns that can illuminate the device under test (DUT). The DUT is setup so that it can be rotated in the azimuth plane around its axis of symmetry. (**B**) Setup for the unfolded state $\psi =0^{\circ }$. (**C**) Setup for a folded state $\psi =30^{\circ }$.

**Figure 10:**
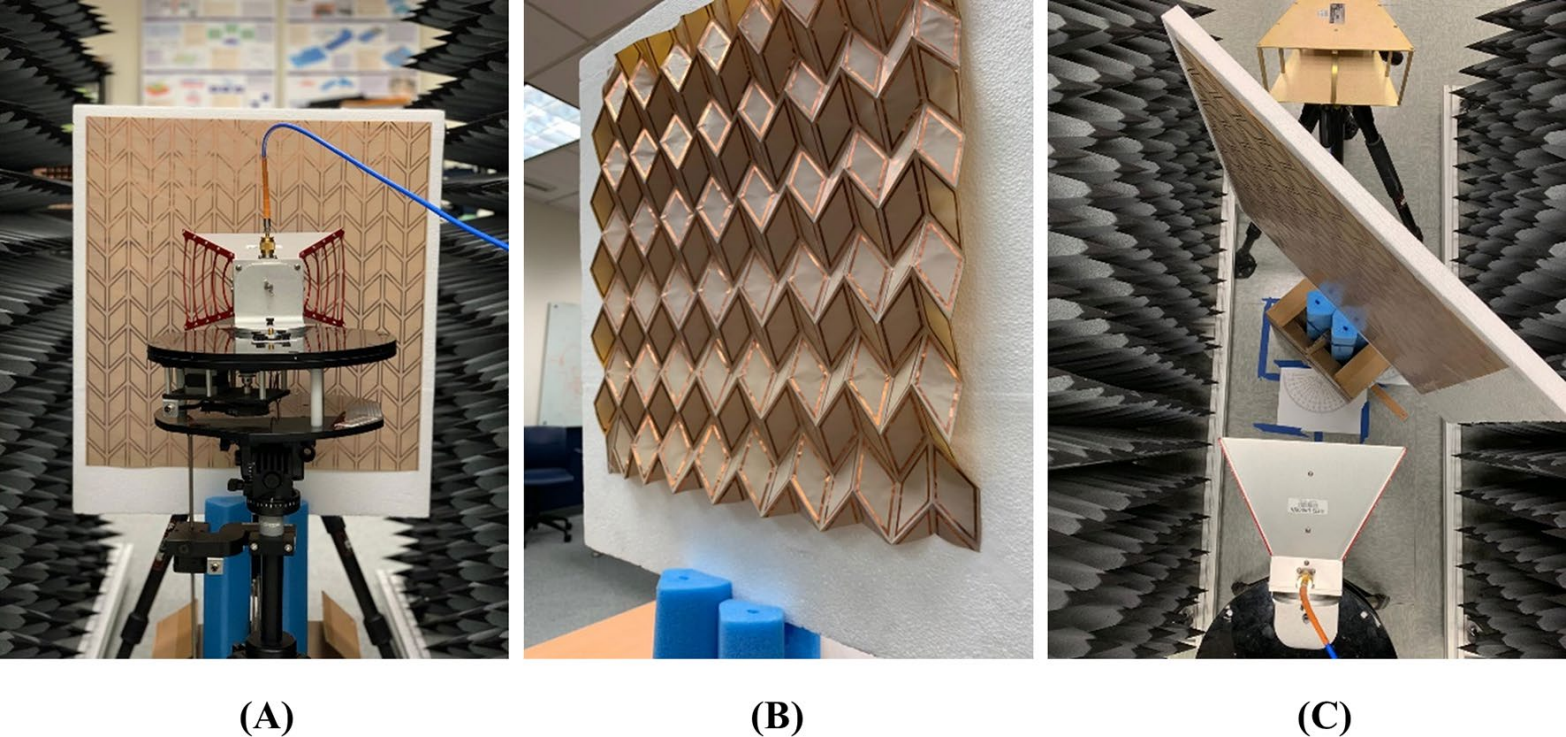
Close view of the FSS measurement setup. (**A**) TE mode setup for $\psi =0^{\circ }$ folding. (**B**) Perspective view of the prototype with $\psi =30^{\circ }$ folding. (**C**) TM mode setup for $\psi =0^{\circ }$ folding and for $45^{\circ }$ incident angle.

The dimensions were appropriately chosen so that a large FSS can be created that minimizes the effect of edge diffraction and spillover. The thickness of the substrate was thoroughly investigated to ensure our design provides optimal mechanical performance when it is folded, i.e., reduce stresses to avoid cracking. It was found that a substrate thickness of $(50{-}120\,\upmu {\hbox {m}})$ provided the best performance when folded. Notably, the thicker the substrate is the more stable and rigid the structure becomes. However, this comes at the expense of stiffness at the creases, which eventually can introduce cracks in the substrate after several folding-unfolding cycles. The FSS was measured in our lab using two horn antennas (an ETS Lindgren 3115 that operates in the range 0.75–18 GHz, and a SAS-571 that operates in the range 0.70–18 GHz), two absorbing walls that operate at the 0.6–18 GHz frequency range and a four port N5222B series PNA network analyzer that operates in the frequency range 10 MHz–26.5 GHz (Figs. 9, 10). Figure 9 presents our measurement setup. The schematic diagram in Fig. 9A demonstrates the connection of the two horns (transmitter–receiver) with the PNA, while the FSS is free to rotate azimuthally on its own axis. The ETS Lindgren horn was used as a transmitter and the SAS-571 Double-Ridge Guide horn as a receiver. The transmitter and receiver can be interchanged. To ensure that the FSS provides a performance that is similar to the one of an infinite array, the distance of the two horns was chosen to be $1\lambda$ (300 m) at the frequency of 1 GHz. Figure 10A,B,C show closer view of measurement setup.

**Figure 11:**
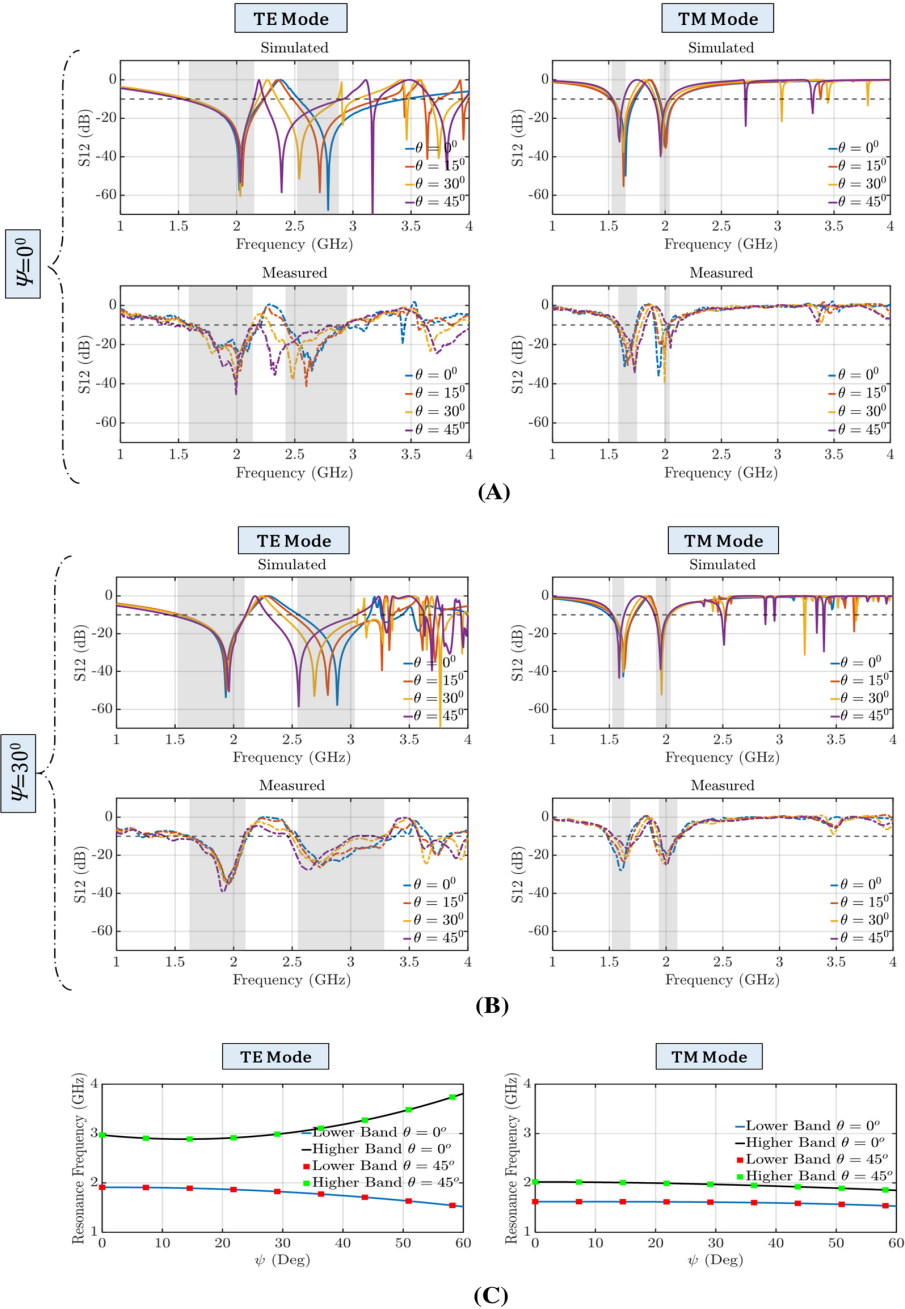
Simulated and measured transmission coefficient for TE and TM polarizations. The horizontal dash line corresponds to $-\,10\,{\hbox {dB}}$ while the shaded areas signify the stopband frequency ranges of the FSS. The dual-band performance is maintained for different folding angles and for impinging waves with different incident angles ($\theta$). (**A**) FSS is flat $\psi =0^{\circ }$. (**B**) FSS is folded $\psi =30^{\circ }$. (**C**) Variation of the FSS’s resonant frequencies (i.e., center frequencies of the two stopbands) versus the folding angle, $\psi$.

Figure 11 shows the simulated and measured FSS’s transmission coefficient at its flat $(\psi = 0^{\circ })$ and folded $(\psi = 30^{\circ })$ states for TE and TM polarized waves with different angles of incidence $(0^{\circ }, 15^{\circ }, 30^{\circ },45^{\circ })$. These results clearly illustrate the dual-band performance of this FSS, which is observed in both measurements and simulations. Measurements and simulations are in good agreement. Specifically, the shaded areas in Fig. 11 highlight the FSS’s stopband frequency range (a stopband is defined as the frequency range where the transmission coefficient is less than $-10\,{\hbox {dB}}$) that remain intact as the wave’s incident angle varies from $0^{\circ }$ to $45^{\circ }$. We also measured this FSS at a folded state to illustrate that this FSS can reconfigure its performance while maintaining its dual-band behavior through folding (see Fig. 11B). As Fig. 11B shows, when the Miura-Ori FSS is folded at $\psi = 30^{\circ }$, the two frequency stopbands move in opposite directions for the case of TE waves, whereas the stopbands move in the same direction and towards lower frequencies for the case of TM waves. Figure 11C shows the variation of the FSS’s resonant frequencies (i.e., center frequencies of the two stopbands) versus the origami structure’s folding angle.

## Discussion

Origami FSSs and other electromagnetic structures with tunable properties have been previously proposed and studied by various researchers^23,42–46^. Also, it has been proven that the mechanical and EM performance of origami designs can be changed by varying their folding angle. However, before this work, it had not been clear how to properly design/select a pattern to achieve a certain electromagnetic behavior. In this work, not only we reveal a new phenomenon, but we also prove that enhanced electromagnetic performance can be achieved by utilizing an origami configuration with appropriately oriented electromagnetic resonators. Specifically, by conforming strongly coupled resonators to a Miura-Ori pattern, a single-band FSS is transformed to a dual-band one. This finding informs us how to properly design an origami pattern to meet a certain target performance. For our specific case of a frequency selective surface, we know that resonators must be closely spaced so they can resonate as a spatial filter^27^. Also, as we showed with our analysis, the resonators must be periodically and spatially modulated so that they can introduce dual-band behavior (see Fig. 2E,D). Specifically, as we change the angle $\gamma$ of the origami pattern (see Fig. 2A,B), the second resonance appears and disappears. This phenomenon is explained in our manuscript through the appearance of the resonances and anti-resonances between the coupled resonators. This finding brings us to the realization that origami EM tessellations (i.e., designs that modulate periodically spaced resonators) can introduce new phenomena by appropriately exciting resonances and anti-resonances. The choice of the origami pattern will determine the EM performance (e.g., if an FSS’s performance will be single-band or multi-band). However, the exploration of different patterns and corresponding resonator designs is out of the scope of this work. We expect though that novel electromagnetic structures, such as FSS, phased arrays, polarization converters, absorbers, can be developed by conforming EM periodic structures to various origami patterns and tessellations.

### Methods

We fabricated the single-layer Miura-Ori-FSS using polyimide substrate of thickness $50\,\upmu {\hbox {m}}$ and of $\epsilon _r=3.4$ and $\mu _r=1$. Two identical prototypes of size $60\times 56.6\,{\hbox {cm}}^2$ were fabricated using a traditional PCB fabrication method. Each prototype consists of 48 unit cells. The Miura-Ori-FSS was manually folded at $\psi =30^{\circ }$. To measure the FSS the prototype was mounted on a foam-board of $\epsilon _r=1.03$. Both the flat and folded states were measured for different angles of incidence by rotating the FSS in the azimuth plane, around its axis of symmetry (Fig. 9C).

## Supplementary Information

Supplementary Movie.
